# Residual reserve index modifies the effect of amyloid pathology on fluorodeoxyglucose metabolism: Implications for efficiency and capacity in cognitive reserve

**DOI:** 10.3389/fnagi.2022.943823

**Published:** 2022-08-12

**Authors:** Cathryn McKenzie, Romola S. Bucks, Michael Weinborn, Pierrick Bourgeat, Olivier Salvado, Brandon E. Gavett

**Affiliations:** ^1^School of Psychological Science, The University of Western Australia, Perth, WA, Australia; ^2^Australian e-Health Research Centre, Commonwealth Scientific and Industrial Research Organisation (CSIRO) Health and Biosecurity, Brisbane, QLD, Australia; ^3^Data61, Commonwealth Scientific and Industrial Research Organisation (CSIRO), Sydney, NSW, Australia

**Keywords:** cognitive reserve, Alzheimer’s disease, amyloid cascade hypothesis, executive function, AT(N) classification system, cognitive resilience, FDG metabolism

## Abstract

**Background:**

The residual approach to measuring cognitive reserve (using the residual reserve index) aims to capture cognitive resilience conferred by cognitive reserve, but may be confounded by factors representing brain resilience. We sought to distinguish between brain and cognitive resilience by comparing interactions between the residual reserve index and amyloid, tau, and neurodegeneration [“AT(N)”] biomarkers when predicting executive function. We hypothesized that the residual reserve index would moderate at least one path from an AT(N) biomarker to executive function (consistent with cognitive resilience), as opposed to moderating a path between two AT(N) biomarkers (suggestive of brain resilience).

**Methods:**

Participants (*N* = 332) were from the Alzheimer’s Disease Neuroimaging Initiative. The residual reserve index represented the difference between observed and predicted memory performance (a positive residual reserve index suggests higher cognitive reserve). AT(N) biomarkers were: CSF β-amyloid_1–42_/β-amyloid_1–40_ (A), plasma phosphorylated tau-181 (T), and FDG metabolism in AD-specific regions ([N]). AT(N) biomarkers (measured at consecutive time points) were entered in a sequential mediation model testing the indirect effects from baseline amyloid to executive function intercept (third annual follow-up) and slope (baseline to seventh follow-up), via tau and/or FDG metabolism. The baseline residual reserve index was entered as a moderator of paths between AT(N) biomarkers (e.g., amyloid-tau), and paths between AT(N) biomarkers and executive function.

**Results:**

The residual reserve index interacted with amyloid pathology when predicting FDG metabolism: the indirect effect of amyloid → FDG metabolism → executive function intercept and slope varied as a function of the residual reserve index. With lower amyloid pathology, executive function performance was comparable at different levels of the residual reserve index, but a higher residual reserve index was associated with lower FDG metabolism. With higher amyloid pathology, a higher residual reserve index predicted better executive function via higher FDG metabolism.

**Conclusion:**

The effect of the residual reserve index on executive function performance via FDG metabolism was consistent with cognitive resilience. This suggests the residual reserve index captures variation in cognitive reserve; specifically, neural efficiency, and neural capacity to upregulate metabolism to enhance cognitive resilience in the face of greater amyloid pathology. Implications for future research include the potential bidirectionality between neural efficiency and amyloid accumulation.

## Introduction

As the global population ages, the number of individuals affected by dementia is expected to grow rapidly (Livingston et al., 2020). Alzheimer’s disease is the leading cause of dementia, and is a major contributor to global disease burden (Nichols et al., 2019). Although the search for disease-modifying treatments has produced some promising candidates (van Bokhoven et al., 2021), prevention is key to reducing the burden of Alzheimer’s disease, and requires an understanding of the biological and environmental factors that confer resistance and resilience across the Alzheimer’s disease continuum (Hampel et al., 2019). “Resistance” refers to the reduced or delayed development of neuropathology (e.g., resistance to Alzheimer’s disease pathology), whereas “resilience” refers to the capacity for the brain to cope with neuropathology (Arenaza-Urquijo and Vemuri, 2020; Stern et al., 2022). For example, high resilience may be reflected by lower than expected neurodegeneration at a given level of Alzheimer’s disease pathology (which we will describe as “brain resilience”), or better than expected cognitive performance at a given level of neurodegeneration (which we will describe as “cognitive resilience”).

A wealth of research shows that modifiable behavioral factors (e.g., education and engagement in cognitively stimulating activities) enhance cognitive resilience in the face of Alzheimer’s disease neuropathology, which then delays or prevents dementia (Livingston et al., 2020; Scheltens et al., 2021). Cognitive reserve is one hypothesized mechanism underlying this cognitive resilience: for a given degree of Alzheimer’s disease neuropathology, an individual with greater cognitive reserve is expected to have better cognitive functioning and show less impaired clinical status compared to an individual with lower cognitive reserve (Stern, 2009; Stern et al., 2022). Although there is substantial support for the theory of cognitive reserve, efforts to conceptualize, operationalize, and understand cognitive reserve are ongoing (Stern et al., 2020).

One approach to measuring cognitive reserve is the residual approach, which operationalizes cognitive reserve as the variance in episodic memory performance that is not explained by demographics and brain integrity, i.e., the difference between observed performance and the performance expected based on demographics and brain integrity. In this way, it may also be considered a measure of the cognitive resilience an individual is expressing at the time of measurement. Compared to proxy measurements of cognitive reserve (e.g., years of education), the residual approach offers a more direct estimate of cognitive reserve and can be measured dynamically (Reed et al., 2010; Bettcher et al., 2019).

Prior research has supported the validity of the residual (hereafter referred to as the residual reserve index) as a measure of cognitive reserve; e.g., a higher residual reserve index has been associated with better cognitive performance, slower cognitive decline, and reduced risk for dementia (Bocancea et al., 2021). Importantly, the residual reserve index has been shown to modify the relationship between neuropathology and separate cognitive or clinical outcomes (e.g., Zahodne et al., 2013), which is a key characteristic of cognitive reserve (Stern et al., 2020). Our previous work showed that the residual reserve index interacted with CSF biomarkers of Alzheimer’s disease: it was positively associated with cognitive decline when biomarkers were positive for Alzheimer’s disease, but there was no association with cognitive decline when CSF biomarkers were consistent with typical aging (McKenzie et al., 2020). This suggests that cognitive reserve, insofar as it is captured by the residual approach, requires a certain degree of Alzheimer’s disease neuropathology before is it “activated” as a predictor of attenuated cognitive decline.

The residual reserve index contains that which is unexplained, or unknown, about cognitive performance. As cognitive performance is ultimately the product of brain function, the unexplained variance in the residual reserve index may be attributed to “unmeasured” brain factors (Mungas et al., 2021). which we assume represent cognitive reserve, to some degree (Reed et al., 2010). This assumption may be a limitation of the residual approach, as the residual reserve index may be confounded by unmeasured brain factors that are not directly related to cognitive reserve (Stern, 2021). For example, brain reserve and brain maintenance may protect cognitive function as a downstream effect of the brain’s resistance or resilience to neuropathology (Nyberg et al., 2012; Stern et al., 2020), rather than the cognitive resilience to neuropathology imparted by cognitive reserve. Compared to cognitive reserve, brain reserve and maintenance are proposed to have different associations with biological and environmental determinants, and to interact differently with biomarkers of Alzheimer’s disease neuropathology to predict better clinical outcomes (Habeck et al., 2016; Cabeza et al., 2018), making them distinct research concepts in Alzheimer’s disease prevention (Stern et al., 2020). Potential contamination of the residual reserve index by factors such as brain reserve or brain maintenance is therefore an important issue that, to our knowledge, is yet to be resolved.

**FIGURE 1:**
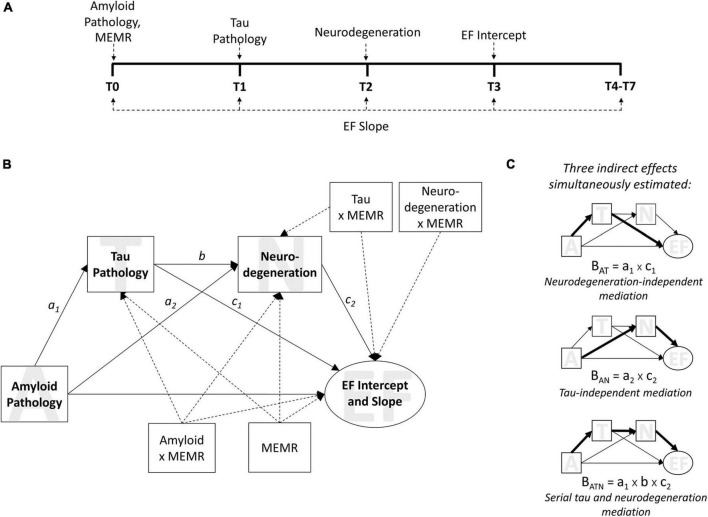
The moderated sequential mediation model. Biomarkers used were the ratio of CSF β-amyloid_1–42_ to β-amyloid_1–40_ (Aβ_42_/Aβ_40_) for amyloid pathology; plasma phosphorylated tau 181 (p-tau181) for tau pathology; and uptake of ^18*F*^fluorodeoxyglucose tracer (FDG metabolism) in AD-specific regions of interest for neurodegeneration. Executive function (EF) was measured using ADNI’s composite measure of EF. MEMR denotes the residual reserve index. **(A)** the timeline of data collection for variables used in the sequential mediation model, from baseline (T0) through to the seventh annual follow-up (T7). **(B)** The moderated sequential mediation model used to test this study’s hypotheses. EF intercept and slope were latent growth factors; the intercept was estimated at T3, and the slope was estimated using all available time points (see Supplementary Figure 2). MEMR was entered as a moderator of all individual paths in the mediation component of the model. Dashed arrows are used for illustrative purposes, to signify paths that relate to moderation by MEMR; solid arrows relate to mediation relationships. Not shown: demographic variables of baseline age, sex, and education were entered as covariates of ADNI-EF intercept and slope. **(C)** The indirect effects estimated in **(B)**. The magnitude of an effect (i.e., B_*AT*_, B_*ATN*_, B_*AN*_) is the product of the *a*, *b*, or *c* paths shown in **(B)**.

The present study sought to address this issue and further test the validity of the residual reserve index as a measure of cognitive reserve by (1) locating its protective effects along an Alzheimer’s disease pathological sequence, and (2) evaluating whether this location reflects a protective mechanism consistent with cognitive reserve theory. To do this, we tested the interactions between the residual reserve index and biomarkers of Alzheimer’s disease pathology and neurodegeneration within a model based on the modified amyloid cascade hypothesis (Figure 1; Jack et al., 2013) and informed by the AT(N) classification system for Alzheimer’s disease biomarkers (Jack et al., 2016). Longitudinal executive function performance was chosen as the distal cognitive outcome, given it is sensitive to decline in both aging and AD (Buckner, 2004; Puente et al., 2015), and separate from the memory composite used to create the residual reserve index (Reed et al., 2010). AT(N) biomarkers and longitudinal executive function outcomes were measured sequentially (Figure 1A) and entered in a mediation model derived from the modified amyloid cascade hypothesis (Figure 1B). The residual reserve index was measured at baseline (T0) and an interaction term was calculated with each biomarker (Figure 1B). By comparing the interactions between the residual reserve index and AT(N) biomarkers at each stage of this pre-defined Alzheimer’s disease pathological sequence, we can test whether the residual reserve index positively predicts downstream executive function performance, and whether the location of its moderating effect is consistent with cognitive reserve theory.

Our first hypothesis was that the effect of amyloid pathology on downstream executive function performance would be serially mediated by tau pathology and neurodegeneration. This hypothesis would be supported by a significant indirect effect of amyloid pathology on executive function performance (intercept and/or slope) via tau pathology and, subsequently, neurodegeneration (Figure 1C, bottom). Support for this hypothesis would affirm that our model of the modified amyloid cascade hypothesis (Jack et al., 2013, 2018) is appropriate in this sample, and provides a basis for testing more specific hypotheses about the role of cognitive reserve within the AT(N) framework.

The second hypothesis was that the residual reserve index would preferentially moderate at least one path from an AT(N) biomarker to executive function performance, as opposed to primarily moderating a path between two biomarkers. This hypothesis would be supported by a significant interaction (or, if more than one interaction is significant, an interaction of greatest magnitude) between the residual reserve index and an antecedent biomarker when predicting executive function performance. Support for this hypothesis would suggest the residual reserve index attenuates the effect of one or more AT(N) biomarkers on cognitive performance (consistent with cognitive resilience), rather than attenuating the effect of an upstream AT(N) biomarker on a downstream AT(N) biomarker (consistent with brain resilience or resistance). For example, if the residual reserve index primarily moderates the influence of neurodegeneration on executive function performance, this would further validate the residual reserve index as a measure of cognitive reserve, by indicating it captures variance in cognitive resilience to neuropathology. A contrasting example might be if the residual reserve index primarily moderates the influence of amyloid pathology on tau pathology; this may suggest that the residual reserve index is capturing a brain resistance mechanism consistent with brain maintenance, instead of (or in addition to) cognitive reserve.

## Materials and methods

Data used in this study were obtained from the Alzheimer’s Disease Neuroimaging Initiative (ADNI) database at http://adni.loni.usc.edu/data-samples/access-data/. ADNI was launched in 2003 as a public-private partnership, and is led by Principal Investigator Michael W. Weiner, MD. The goal of ADNI is to improve understanding of the progression of mild cognitive impairment and early Alzheimer’s disease, by combining biological markers, e.g., magnetic resonance imaging (MRI), with clinical and neuropsychological assessment. For more information, please see http://adni.loni.usc.edu/.

### Participants

Data were collected by ADNI investigators at 59 sites in North America. For maximum data coverage, the current study used data from the ADNI1, ADNIGO, and ADNI2 phases (*N* = 2,351 participants). For inclusion in ADNI, participants needed to be aged between 55 and 90 years, in generally good health, and willing to participate in a longitudinal study that included neuroimaging and collection of blood and CSF biomarkers. Full inclusion criteria can be found at http://adni.loni.usc.edu/. Written informed consent was obtained from each participant, per the research ethics requirements at each participating ADNI site.

### Neuroimaging biomarkers

Baseline measures of hippocampal, whole brain, white matter hyperintensity, and total intracranial volume, all derived from MRI, were used to create the residual reserve index (Reed et al., 2010). Details of ADNI’s neuroimaging protocols have been described previously (Jack et al., 2008) and can be downloaded from http://adni.loni.usc.edu/. Pre-processed T1-weighted MP-RAGE scans obtained from 3.0-Tesla scanners were downloaded from the ADNI database. The volumes were processed using the longitudinal pipeline in Freesurfer version 6.0.0.^1^ Total intracranial volume was estimated using an atlas-based spatial normalization procedure in Freesurfer (Buckner et al., 2004).

Pre-processed WMH volumes, obtained from T2-weighted FLAIR scans, were downloaded directly from the ADNI database. The distribution of WMH volumes was strongly positively skewed; therefore, this variable was log-transformed before analyses.

The mean ^18*F*^fluorodeoxyglucose (FDG) standard uptake value ratio across five meta-regions of interest (normalized using the pons as a reference) was used as a measure of neurodegeneration (Jack et al., 2018). Details of ADNI’s FDG PET acquisition methods are available at http://adni.loni.usc.edu/ and have been described previously (e.g., Landau et al., 2010).

### Fluid biomarkers

The ratio of β-amyloid_1–42_ to β-amyloid_1–40_ (Aβ_42_/Aβ_40_) in CSF was used as the measure of amyloid pathology (Jack et al., 2018); lower Aβ_42_/Aβ_40_ values indicate greater amyloid pathology. Baseline (T0) Aβ_42_/Aβ_40_ measurements were downloaded directly from the ADNI database. The ADNI CSF data collection and Aβ_42_/Aβ_40_ analysis methods have been detailed previously (Korecka et al., 2014; Kang et al., 2015). Previous work has shown that, compared to Aβ_42_ concentration alone, CSF Aβ_42_/Aβ_40_ is a more specific measure of Alzheimer’s disease pathology, and is a more robust proxy for cortical Aβ_42_ deposition (Lewczuk et al., 2014, 2017; Janelidze et al., 2016), possibly because Aβ_42_/Aβ_40_ controls for individual variation in total β-amyloid peptides, and is less susceptible to confounding factors (Blennow and Zetterberg, 2018).

Tau pathology was measured using plasma phosphorylated tau 181 (p-tau181; Jack et al., 2018). Data from baseline (T0) and the first annual follow-up (T1) were downloaded directly from the ADNI database to be used in the decomposition model and moderated sequential mediation model, respectively. We considered using CSF p-tau181 as our tau biomarker, but decided to use plasma to capitalize on the larger sample size at T1, per ADNI protocol (Kang et al., 2015). Recent studies support the validity of plasma p-tau181 as an Alzheimer’s disease-specific biomarker of cortical tau aggregation and a predictor of longitudinal clinical outcomes (Janelidze et al., 2020; Karikari et al., 2021; Moscoso et al., 2021; Palmqvist et al., 2021).

### Neuropsychological data

ADNI’s composite memory measure, ADNI-Mem (Crane et al., 2012), was used as the source of variance to be decomposed to create the baseline (T0) residual reserve index (Figure 1). ADNI-Mem is derived using all available memory tasks, such as the Rey Auditory Verbal Learning Test (Rey, 1964), and has good validity as a predictor of conversion from MCI to dementia in the ADNI cohort (Crane et al., 2012).

ADNI’s composite executive function measure, ADNI-EF (Gibbons et al., 2012), was used to model the growth factors (intercept and linear slope) used as distal outcome variables in the moderated sequential mediation. A composite of executive function measures, e.g., Digit Span Backward and Digit Symbol Substitution from the Wechsler Adult Intelligence Scale 3rd edition (Wechsler, 1997), ADNI-EF is more sensitive to change over time in the ADNI sample than its constituent tests (Gibbons et al., 2012). ADNI-EF scores were standardized according to the sample mean and standard deviation at T0, so that change in ADNI-EF scores can be interpreted as change from the T0 mean, in T0 standard deviation units.

Demographic variables were also used in the analyses. These included years of education (full-time equivalent; centered on 12 years) and number of apolipoprotein-E ε4 alleles (APOE4 status; range 0–2), and dichotomous variables representing sex (1 = male, 0 = female), race (1 = African American, 0 = not African American), and ethnicity (1 = Hispanic, 0 = non-Hispanic). Demographic variables were used to define the residual reserve index. Education, sex, and baseline age (centered on the sample average 73.10 years) were also included as covariates of the ADNI-EF intercept and slope.

### Statistical analyses

**FIGURE 2:**
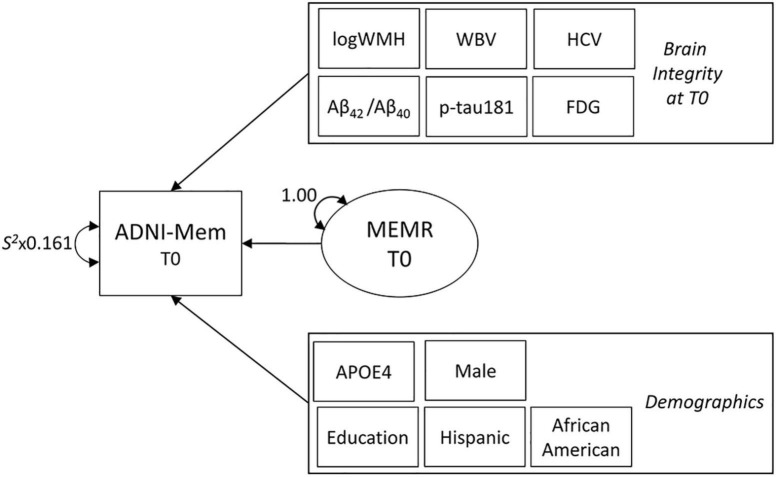
The decomposition model used to define the residual reserve index. Variance in baseline (T0) episodic memory performance (ADNI-Mem) is decomposed into variance explained by indicators of brain integrity (also measured at T0), demographic indicators, and the residual reserve index (MEMR). Rectangles represent observed variables and ovals represent latent variables. Observed brain integrity and demographic variables are contained within single rectangles to simplify the diagram. Parameters are freely estimated unless labeled otherwise. Double-ended arrows represent variance or residual variance. *S*^2^ = sample variance (multiplied by measurement error). Not shown: whole brain (WBV) and bilateral hippocampal (HCV) volumes were regressed onto total intracranial volume to correct for head size. Correlations between observed variables were initially freely estimated, then non-significant correlations were constrained to zero to facilitate model convergence. Correlations between MEMR and observed variables were constrained to zero. ADNI-Mem, ADNI’s composite of episodic memory performance; logWMH, log-transformed white matter hyperintensity volume; Aβ_42_/Aβ_40_, ratio of CSF β-amyloid_1–42_ to β-amyloid_1–40_; p-tau181, plasma phosphorylated tau 181; FDG, uptake of ^18*F*^fluorodeoxyglucose tracer in AD-specific regions of interest; APOE4, number of apolipoprotein-E ε4 alleles.

Analyses were performed in Mplus version 8 (Muthén and Muthén, 2017) using two structural equation models (SEMs): (1) the decomposition of episodic memory performance to create the residual reserve index (Figure 2), and (2) the testing of our hypotheses using a moderated sequential mediation model (Figure 1) based on the modified amyloid cascade hypothesis (Jack et al., 2013, 2018). Although it is possible to test moderated mediation models involving latent variables in Mplus, estimating the decomposition and moderated sequential mediation models simultaneously was too computationally intensive due to the number of latent variable interactions involved. Instead, we used factor scores from the decomposition model as observed variables in the moderated sequential mediation model. Given that saved factor scores can be complicated by the presence of factor score indeterminacy, which may be a source of error in subsequent analyses (Rigdon et al., 2019), we used Bayesian analysis to impute 30 sets of plausible values for the residual reserve index, to use in the subsequent moderated sequential mediation model (Asparouhov and Muthén, 2010).

Maximum likelihood (ML) was the primary method used to estimate the decomposition and moderated sequential mediation models; the fit of ML models was evaluated based on converging evidence from the comparative fit index (CFI), Tucker-Lewis Index (TLI), root mean square error of approximation (RMSEA), and the standardized root mean square residual (SRMR), using standard criteria (Hu and Bentler, 1999). Plausible values from the decomposition model were imputed and saved using Bayesian SEM. The reliability of the plausible values was estimated with intraclass correlation using the “psych” package (Revelle, 2021) in R studio version 2021.09.0 (RStudio Team, 2021) and R version 4.0.4 (R Core Team, 2021).

#### The decomposition model for the residual reserve index

A structural equation model (Figure 2), adapted from Reed et al. (2010), was used to decompose variance in ADNI-Mem performance into variance explained by demographic variables, variance explained by biomarkers of brain integrity, and a latent variable containing residual variance (MEMR; the residual reserve index). MEMR represents the variance in memory performance that is not explained by brain integrity and demographics. A large, positive residual indicates an individual is performing better than expected based on their demographics and brain integrity; such an individual would be interpreted as having high cognitive reserve.

To ensure MEMR represents a unique component of ADNI-Mem variance, its correlations with all other observed variables in the model were constrained to zero. Reed et al.’s (2010) original model, non-significant correlations between observed variables were fixed to zero to facilitate model convergence. Demographic variables that were small, non-significant predictors of ADNI-Mem were removed to increase parsimony in the subsequent Bayesian analysis. Non-significant brain biomarker variables were not removed: because the concept of “unmeasured” brain features prominently in the interpretation of the residual reserve index (Mungas et al., 2021), it was considered more important to ensure all baseline biomarkers of brain pathology and neurodegeneration were accounted for in the decomposition model.

#### Bayesian imputation of plausible values

The decomposition model (Figure 2), identified using ML estimation, was run using Bayesian estimation with Markov Chain Monte Carlo sampling. The criterion for convergence was a potential scale reduction factor that stayed below 1.1 for the second 50% of iterations, and remained stable when the total number of iterations was doubled (Gelman and Rubin, 1992; Muthén and Asparouhov, 2011). Fit was evaluated against the criterion of a posterior predictive *p*-value (PPP) > 0.05 (Muthén and Asparouhov, 2011). ML-derived starting values were entered into the Bayesian model, with one exception necessary to facilitate model fit: whereas the residual variance of ADNI-Mem was fixed to our previously calculated estimate of measurement error of 0.161 (McKenzie et al., 2020) in the ML model, it was freely estimated in the Bayesian model, and a highly informative prior [*N*(0.161, 0.00002)] was specified instead.

After achieving acceptable convergence and fit, 30 imputations of Bayesian plausible values for the residual reserve index were saved. The reliability of the plausible values was determined using the ICC(2,*k*); i.e., the ICC based on a mean-rating, absolute-agreement, 2-way random effects model (Shrout and Fleiss, 1979; McGraw and Wong, 1996; see Supplementary material for more information).

#### The moderated sequential mediation model

This study’s hypotheses were tested using the moderated sequential mediation model shown in Figure 1. This model is based on Andrew Hayes’ PROCESS Model 92 (Hayes, 2017); Mplus syntax was adapted from syntax published by Hayes (2015) and Stride et al. (2015). Results from the 30 imputed datasets were aggregated in Mplus; parameter estimates were pooled across imputations, and standard errors were calculated using the within-imputation standard errors and between-imputation variance in parameter estimates (Schafer, 1997; Muthén and Muthén, 2017). As bootstrapped confidence intervals are not available when analyzing multiple imputed datasets in Mplus, the significance of the mediation effects was evaluated by entering the average plausible values into the moderated mediation model and obtaining bootstrapped bias-corrected 95% confidence intervals for the indirect effects.

Amyloid pathology was modeled as a predictor of ADNI-EF performance (intercept and slope) directly, and indirectly via tau pathology and neurodegeneration mediators. The ADNI-EF intercept was defined by data from the third annual follow-up (T3), i.e., 1 year after neurodegeneration (T2 FDG metabolism) was measured—and the ADNI-EF slope was defined using annual data from T0 to the seventh follow-up (T7). Age, years of education, and sex were entered as covariates of the ADNI-EF intercept and slope. Three indirect effects (Figure 1C) were tested simultaneously within the moderated sequential mediation model: (1) Amyloid pathology → mtau pathology → aneurodegeneration eurode-EF performance (B_*ATN*_); (2) Amyloid pathology → mtau pathology → au pa-EF performance (B_*AT*_); and (3) Amyloid pathology myneurodegeneration → eurod-EF performance (B_*AN*_). B_*ATN*_ tests the serial mediation pathway predicted by the amyloid cascade hypothesis (Jack et al., 2013, 2018). B_*AT*_ tests a mediation pathway that is independent from neurodegeneration; it reflects the degree to which tau pathology mediates the effect of amyloid pathology on ADNI-EF performance via mechanisms other than neurodegeneration. B_*AN*_ tests a mediation pathway that controls for tau pathology; it reflects the degree to which neurodegeneration mediates the effect of amyloid pathology on ADNI-EF performance via mechanisms independent from tau pathology.

To examine the moderating effects of the residual reserve index, interactions between MEMR and the biomarkers were also tested simultaneously within the model (as shown in Figure 1B). These interactions were entered as predictors of tau pathology, neurodegeneration, and ADNI-EF intercept and slope, to test our hypothesis that the residual reserve index would preferentially moderate a path from an antecedent biomarker to ADNI-EF performance, rather than a path between two AT(N) biomarkers. If a significant interaction was found, the influence of this interaction on the relevant indirect pathway(s) (B_*ATN*_, B_*AT*_, and/or B_*AN*_) would be quantified using the index of moderated mediation and by plotting conditional indirect effects (Hayes, 2015).

## Results

Of the 2,351 participants whose data were used to create the residual reserve index, data from 332 participants were used in the moderated sequential mediation model. This was the number of participants with non-missing data for amyloid, tau, and neurodegeneration biomarkers. Participant characteristics for the total sample and moderated sequential mediation sample are presented in Table 1.

**TABLE 1:** Participant characteristics.

Variable	Total sample (*N* = 2,351)	Moderated sequential mediation sample (N = 332)	Difference^a^
**Age (years)**			
*M* (*SD*)	73.10 (7.27)	72.11 (7.52)	*t*(2,346) = 2.663*
**Sex**			
N (%) male	1,244 (52.90)	179 (53.92)	*X*^2^(1) = 0.14
**Race/ethnicity**			
N (%) African American	137 (5.80)	7 (2.11)	*X*^2^(1) = 9.39*
N (%) Hispanic	105 (4.50)	7 (2.11)	*X*^2^(1) = 5.00*
APOE ε4 status			
N (%) 0 alleles	953 (40.50)	200 (60.20)	*X*^2^(2) = 8.38*
N (%) 1 allele	665 (28.30)	103 (31.00)	
N (%) 2 alleles	176 (7.50)	29 (8.70)	
**Education (years)**			
*M* (*SD*)	16.06 (2.75)	16.39 (2.57)	*t*(2,346) = 2.39*
**Clinical Diagnosis (T0)**			
N (%) CN	849 (36.10)	101 (30.40)	*X*^2^(2) = 51.49**
N (%) MCI	1,074 (45.70)	208 (62.70)	
N (%) AD	407 (17.30)	23 (6.9)	
N (%) missing	21 (0.90)	0 (0.00)	
**ADNI-Mem (T0)**			
*M* (*SD*)	0.31 (0.90)	0.55 (0.77)	*t*(498.73) = 5.81**
**ADNI-EF (T0)**			
*M* (*SD*)	0.27 (1.08)	0.58 (0.94)	*t*(490.94) = 5.74**
**ADNI-EF (T3)**			
*M* (*SD*)	0.24 (1.14)	0.48 (1.05)	*t*(855) = 3.27*
**HCV (T0; mm3)**			
*M* (*SD*)	6989.14 (1130.00)	7074.99 (1058.22)	*t*(1,548) = 1.52
**WBV (T0; mm^3^)**			
*M* (*SD*)	910437(98483.23)	919026.36 (98003.40)	*t*(1,548) = 1.75
**WMH (T0; mm^3^)**			
*M* (*SD*)	6.02 (9.43)	6.16 (9.98)	*t*(1,430) = 0.31
TIV (mm^3^)			
*M* (*SD*) *M* (*SD*)	1514968.61(164049.96)	1513548.84 (156447.16)	*t*(1,548) = 0.17)
**Aβ_42_/ Aβ_40_ (T0)**			
*M* (*SD*)	0.14 (0.06)	0.15 (0.06)	*t*(844) = 3.26*
**p-tau181 (T0; pg/ml)**			
*M* (*SD*)	18.48 (18.83)	17.54 (12.32)	*t*(876) = 1.10
**p-tau181 (T1; pg/ml)**			
***M* (*SD*)**	18.59 (11.56)	16.85 (9.64)	*t*(726.40) = 3.84**
**FDG metabolism (T0)**			
*M* (*SD*)	1.23 (0.15)	1.28 (0.12)	*t*(734.73) = 8.47**
**FDG metabolism (T2)**			
*M* (*SD*)	1.21 (0.17)	1.26 (0.15)	*t*(644.05) = 7.59**

T0 amyloid, p-tau181, MRI, and FDG biomarkers were used to define the residual reserve index (Figure 2); T1 and T2 measurements, respectively, were used in the sequential moderated mediation model (Figure 1B). APOE ε4, apolipoprotein E ε4; CN, cognitively normal; MCI, mild cognitive impairment; AD, Alzheimer’s disease; ADNI-Mem, ADNI’s composite measure of episodic memory; ADNI-EF, ADNI’s composite measure of executive function; HCV, bilateral hippocampal volume; WBV, whole brain volume; WMH, white matter hyperintensity volume; TIV, total intracranial volume; Aβ_42_/Aβ_40_, ratio of CSF β-amyloid_1–42_ to β-amyloid_1–40_ (the biomarker of amyloid pathology; lower values indicate more severe pathology); p-tau181, plasma phosphorylated tau 181 (the biomarker of tau pathology; higher values indicate more severe pathology); FDG, uptake of ^18*F*^fluorodeoxyglucose tracer in AD-specific regions of interest (the biomarker of neurodegeneration).

^*a*^Difference between the participants included in the moderated sequential mediation sample, and the participants excluded due to missing data on predictor variables.

*p < 0.05.

**p < 0.001.

On average, the sample used in the moderated sequential mediation analysis were slightly younger, had a smaller proportion of African American and Hispanic participants, a smaller proportion of individuals with one or more APOE ε4 alleles, slightly higher education, lower amyloid pathology at T0, poorer T0 ADNI-Mem performance, higher T0 and T3 ADNI-EF performance, lower tau pathology at T1, and higher FDG metabolism at T0 and T2. The proportion of individuals diagnosed as cognitively normal or mild cognitive impairment at T0 was also larger in the sample whose data were used in the moderated mediation, and the proportion of individuals diagnosed with Alzheimer’s disease was smaller.

### The decomposition model for the residual reserve index

The decomposition model (Figure 2) fit well using ML estimation: RMSEA = 0.023, 90% CI (0.013–0.035); CFI = 0.997; TLI = 0.990; SRMR = 0.019. Small, non-significant demographic predictors of ADNI-Mem were ethnicity, β = 0.001, *SE* = 0.018, *p* = 0.938, and race, β = 0.009, *SE* = 0.018, *p* = 0.634; these variables were removed to improve convergence of the Bayesian model. Although ethnocultural factors play a significant role in the development and progression of Alzheimer’s disease, the majority of the ADNI cohort is non-Hispanic white (Birkenbihl et al., 2020), which limits our ability to find significant independent effects of race or ethnicity on cognitive performance in this sample.

### Bayesian imputation of plausible values

The Bayesian decomposition model fit well, *PPP* = 0.418. Comparison of the standardized Bayesian parameter estimates with the standardized ML estimates indicated good consistency between the two models (see Supplementary material). High agreement was also demonstrated across the 30 sets of imputed MEMR plausible values, ICC(2, 30) = 0.973, 95% CI (0.972, 0.975).

### Moderated sequential mediation

**FIGURE 3:**
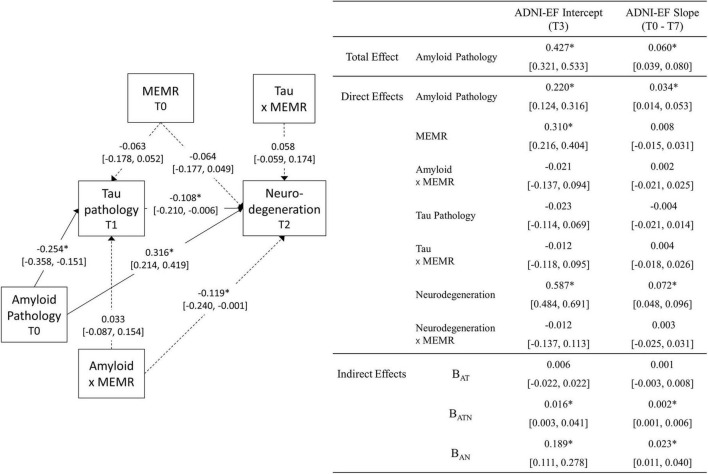
Unstandardized estimates (95% CI) of the parameters obtained from the moderated sequential mediation model (*N* = 332). Parameter estimates that are significant at the *p* < 0.05 level are marked with *. The 95% CIs for the indirect effects (bottom-right of figure) are bias-corrected bootstrapped confidence intervals (2,000 draws), obtained from a model estimated using the average residual reserve index (MEMR) plausible values. All other 95% CIs are symmetric intervals (±1.96 standard errors) obtained by pooling results from 30 imputed datasets. Dashed lines are used for illustrative purposes, to signify paths that relate to the moderating effect of MEMR; solid lines relate to mediation relationships. Biomarkers used were the ratio of CSF β-amyloid_1–42_ to β-amyloid_1–40_ (Aβ_42_/Aβ_40_) for amyloid pathology; plasma phosphorylated tau 181 (p-tau181) for tau pathology; and uptake of ^18*F*^fluorodeoxyglucose tracer (FDG metabolism) in AD-specific regions of interest for neurodegeneration. ADNI-EF denotes ADNI’s composite measure of executive function. B_*AT*_, the amyloid → tau → ADNI-EF indirect effect; B_*ATN*_, the amyloid fect; B neurodegeneration → ADNI-EF indirect effect; B_*AN*_, the amyloid → neurodegeneration → ADNI-EF indirect effect.

Moderation and mediation effects were tested using the moderated sequential mediation model shown in Figure 1B; the results are shown in Figure 3. Model fit was good, RMSEA = 0.030, 90% CI (0.013, 0.043); CFI = 0.982; TLI = 0.980; SRMR = 0.054. The MEMR plausible values were averaged for each subject and entered separately into the moderated sequential mediation model to obtain the bias-corrected bootstrapped (*B* = 2,000) 95% confidence intervals for the indirect effects that are reported in the bottom-right of Figure 3 (Hayes, 2009). Model fit was also good using the average plausible values, RMSEA = 0.046, 90% CI (0.035, 0.057); CFI = 0.964; TLI = 0.960; SRMR = 0.058.

#### Total and direct effects

The total effect of amyloid pathology on ADNI-EF intercept and slope was significant and positive, indicating that lower levels of amyloid pathology were associated with higher predicted ADNI-EF scores at T3, and a slower predicted rate of ADNI-EF decline over 7 years. Approximately half of this total effect remained after accounting for the mediators, indicating a significant direct effect of amyloid on ADNI-EF intercept and slope that was independent of tau pathology and neurodegeneration.

**FIGURE 4:**
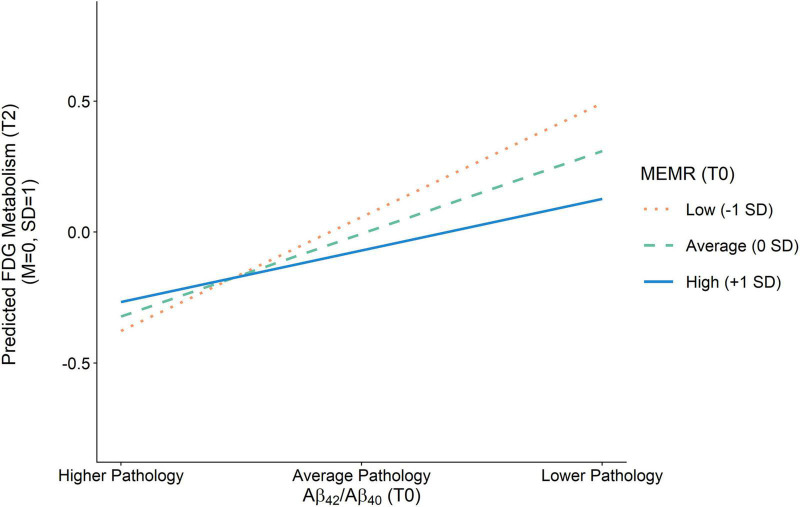
Effect of amyloid pathology on FDG metabolism as a function of the residual reserve index. Amyloid pathology (Aβ_42_/Aβ_40_) was modeled as a continuous variable and is separated into higher pathology (–1 SD), average pathology (0 SD), and lower pathology (+1 SD) for illustration purposes. Aβ_42_/Aβ_40_, ratio of CSF β-amyloid_1–42_ to β-amyloid_1–40_; FDG, uptake of ^18*F*^fluorodeoxyglucose tracer in AD-specific regions of interest; MEMR, the residual reserve index.

Less severe amyloid pathology was also uniquely associated with less severe tau pathology at T1 and less severe neurodegeneration at T2, such that an individual with amyloid pathology that is one standard deviation below the sample mean (i.e., an Aβ_42_/Aβ_40_ value one standard deviation above the mean) would be expected to have an approximately one-third standard deviation reduction in tau pathology and neurodegeneration, respectively. The interaction between amyloid pathology and MEMR was also a significant predictor of neurodegeneration (Figure 4). While higher MEMR was associated with more severe neurodegeneration (i.e., lower FDG metabolism) at less severe levels of amyloid pathology, this relationship was inverted at more severe levels of amyloid pathology, such that higher MEMR was associated with less severe neurodegeneration (i.e., higher FDG metabolism). No other significant MEMR interactions were found.

#### Indirect effects

Of the indirect effects, B_*AN*_ and B_*ATN*_ were significant (Figure 3), indicating that, in this sample, there were three different pathways by which amyloid pathology predicted ADNI-EF intercept and slope: (1) via mechanisms that are independent from downstream tau pathology and neurodegeneration (i.e., direct effects), (2) serially through tau pathology and neurodegeneration (i.e., B_*ATN*_), and (3) through neurodegeneration independent from tau pathology (i.e., B_*AN*_). Given that the only significant MEMR interaction was the interaction between MEMR and amyloid pathology when predicting FDG metabolism (i.e., MEMR moderated the amyloid → FDG metabolism direct effect), the rest of this section will focus on interpreting the conditional B_*AN*_ indirect effects.

##### Conditional amyloid → fluorodeoxyglucose metabolism → executive function effects

**FIGURE 5:**
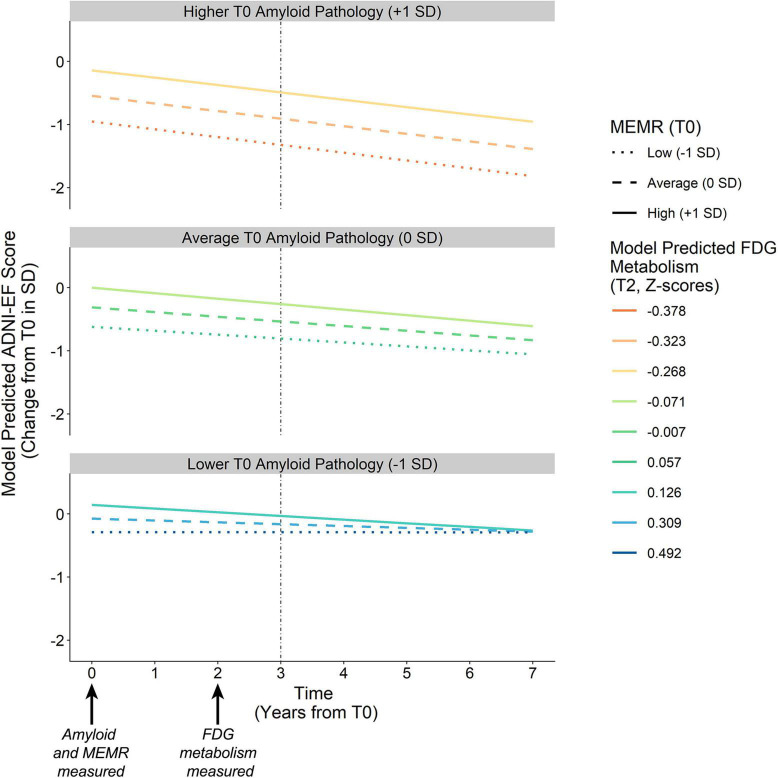
Model-predicted ADNI-EF intercept and slope derived from conditional B_*AN*_ indirect effects. Amyloid pathology (T0) is defined by continuous variable Aβ_42_/Aβ_40_, which is separated into higher pathology (–1 SD), average pathology (0 SD), and lower pathology (+1 SD) for illustration purposes. Colored lines represent the downstream neurodegeneration (i.e., FDG metabolism) predicted by the interaction between amyloid pathology Aβ_42_/Aβ_40_ and the residual reserve index (MEMR) at T0. Solid vs. dashed lines denote the chosen levels of MEMR. The vertical dashed line denotes the ADNI-EF intercept, modeled to succeed measurement of the antecedent biomarkers. B_*AN*_, the amyloid eneurodegeneration → ADNI-EF indirect effect; FDG, uptake of ^18*F*^fluorodeoxyglucose tracer in AD-specific regions of interest; MEMR, the residual reserve index; Aβ_42_/Aβ_40_, ratio of CSF β-amyloid_1–42_ to β-amyloid_1–40_.

To examine how the B_*AN*_ indirect effect depends on MEMR (the residual reserve index), the index of moderated mediation (IMM_*AN*_) was calculated (Hayes, 2015). The index of moderated mediation was significantly different from zero for the ADNI-EF intercept, IMM_*AN*_ = −0.070, 95% bootstrapped CI (−0.158, −0.035), and the ADNI-EF slope, IMM_*AN*_ = −0.009, 95% bootstrapped CI (−0.023, −0.004), indicating that the magnitude of B_*AN*_ was negatively associated with the residual reserve index. Figure 5 illustrates how the interaction between amyloid pathology and the residual reserve index, when predicting neurodegeneration, alters the downstream ADNI-EF outcomes.

Higher MEMR was associated with higher predicted ADNI-EF performance at T3 across all levels of amyloid pathology, although the association between MEMR and the T3 ADNI-EF performance became stronger as amyloid pathology became more severe. MEMR was not associated with change in ADNI-EF slope at average and more severe levels of amyloid pathology. At less severe levels of amyloid pathology, the rate of change in ADNI-EF appeared to depend somewhat on MEMR but, overall, ADNI-EF rate of change was predicted to remain fairly stable at low T0 levels of amyloid burden, regardless of the value of the residual reserve index.

These results suggest that higher residual reserve index values predict relative preservation of future executive function performance, but the mechanism by which this protection happens depends on baseline amyloid pathology. When amyloid pathology at T0 is less severe, a higher T0 residual reserve index relates to relatively *lower* FDG metabolism at T2, and a similar executive function performance at T3 compared to lower values of the residual reserve index. In contrast, when amyloid pathology at T0 is more severe, the protective effect of the residual reserve index at T0 is related to relatively *higher* FDG metabolism at T2 and, subsequently, better executive function performance at T3.

## Discussion

The current study aimed to test the validity of the residual reserve index as a measure of cognitive reserve by (1) locating its protective effects within a sequential mediation model based on the modified amyloid cascade hypothesis of Alzheimer’s disease, and (2) evaluating whether this location reflects a protective mechanism consistent with cognitive reserve theory. Our first hypothesis was supported: the effect of baseline amyloid pathology on future executive function performance was partially serially mediated by tau pathology and neurodegeneration, although the tau-independent (amyloid → mneurodegeneration → eexecutive function) pathway explained the greatest proportion of variance in the total amyloid effect. Our second hypothesis, that the residual reserve index would preferentially moderate at least one path from an antecedent biomarker to executive function performance, was not supported. The only significant interaction was the moderation by the T0 residual reserve index of the path between T0 amyloid pathology and consequent neurodegeneration at T2, which was measured using FDG metabolism. Overall, there was a negative association between amyloid pathology and FDG metabolism, but the magnitude of this effect varied depending on the residual reserve index.

Although the location of the residual reserve index’s strongest interaction effect is inconsistent with our hypothesis, the residual reserve index still showed a downstream protective effect on executive function at T3 (i.e., the executive function intercept) via its association with FDG metabolism, which is consistent with the cognitive resilience that is assumed to be conferred by cognitive reserve. When T0 amyloid pathology was less severe, a higher T0 residual reserve index predicted relatively *lower* T2 FDG metabolism, but there was little difference in predicted T3 executive function performance at varying levels of the residual reserve index. These results suggest that, at less severe levels of amyloid pathology, individuals with a higher residual reserve index are expected to show lower FDG metabolism and a similar level of future executive function performance, relative to those with a lower residual reserve index. At increasingly severe levels of T0 amyloid pathology, a higher residual reserve index predicted higher T2 FDG metabolism, which consequently predicted better T3 executive function.

While a negative association between amyloid pathology and FDG metabolism in AD-affected regions is well established in individuals with Alzheimer’s disease dementia (Mosconi et al., 2009; Nordberg et al., 2010), some studies show a positive association between amyloid burden and FDG metabolism in individuals with MCI, as well as APOE ε4 carriers and amyloid-positive individuals with intact cognition (Cohen et al., 2009; Kadir et al., 2012; Johnson et al., 2014; Oh et al., 2014; Yi et al., 2014; Kemppainen et al., 2015), which may indicate there is a compensatory increase in metabolism early in the Alzheimer’s disease pathological continuum that has disappeared by the time the dementia syndrome manifests. To support the contention that an increase in metabolism is compensatory in nature, the increase should be associated with improved cognitive outcomes (Apostolova et al., 2018), and this was demonstrated in a study by Ossenkoppele et al. (2014), in which amyloid positivity was associated with higher FDG metabolism, which subsequently predicted higher episodic memory performance, in cognitively normal older adults. Our positive association between the residual reserve index and FDG metabolism at higher levels of amyloid pathology could be interpreted as a compensatory upregulation of metabolism since it benefits downstream executive function performance.

If we consider FDG metabolism as a proxy for neurodegeneration (where lower metabolism represents greater neurodegeneration), we might expect that, at a given degree of cognitive function or clinical severity, individuals with higher cognitive reserve will show lower FDG metabolism (indicative of greater neurodegeneration) compared to individuals with lower cognitive reserve, because they are able to maintain cognitive performance in the face of more severe brain changes (Stern et al., 1992). Studies using education as a proxy for cognitive reserve tend to demonstrate this pattern of poorer brain integrity at higher levels of education (Perneczky et al., 2006; Garibotto et al., 2008; Kemppainen et al., 2008). Ewers et al. (2013) found that, while controlling for cognitive status, the association between education and FDG metabolism changed depending on level of amyloid pathology, though they found the opposite pattern to ours: higher education was associated with lower FDG metabolism in amyloid positive participants, and higher FDG metabolism in amyloid negative participants. There are a few possible explanations for our different findings. First, we predicted future executive function performance, rather than controlling for cognitive performance. As such, we are reporting the association between the residual reserve index and FDG metabolism that predicts variability in downstream executive function performance. Second, we did not select our sample based on pathological severity or cognitive performance at baseline, meaning there may be more variation in the expression of cognitive reserve compared to a sample of clinically normal older adults. Third, our results may highlight an important distinction between education and the residual reserve index: whereas education is a static estimate of cognitive reserve over the lifetime, the residual reserve index is intended to capture an individual’s *expression* of cognitive reserve (specifically, the cognitive resilience they are exhibiting) at the time of measurement.

Our findings suggest that the residual reserve index represents variability in neural efficiency and capacity, which are two of the proposed mechanisms underlying cognitive reserve (Barulli and Stern, 2013). Briefly, efficiency is defined as the degree of neural activity required to function, and capacity as the maximum level of neural activity that can be utilized in the face of increasing functional demands or neuronal injury. Relevant to our study is the hypothetical model of cognitive reserve and amyloid accumulation presented by Jagust and Mormino (2011). Based on growing evidence suggesting that higher metabolic activity accelerates amyloid accumulation, the authors proposed that cognitive reserve slows the deposition of cortical amyloid through an association with greater neural efficiency; then, once a threshold of amyloid burden is reached, an increase in neural activity may follow. This increase may be due to an immunological response, or it may reflect compensatory activation to preserve function once neurodegeneration has begun.

The findings of the current study can be interpreted in line with the efficiency and compensatory activity described in Jagust and Mormino’s (2011) model: the negative relationship between the residual reserve index and FDG metabolism at less severe amyloid pathology may represent neural efficiency, and the positive relationship at higher levels of pathology may represent a compensatory response to amyloid burden. This proposed neural efficiency does not show a pronounced benefit on future executive function performance, as T3 performance was comparable at different levels of the residual reserve index; this is consistent with our previous work using ADNI data, which showed that the residual reserve index was not a meaningful predictor of future executive function in Alzheimer’s disease pathology-negative individuals (McKenzie et al., 2020). Nonetheless, these results indicate that a higher residual reserve index is associated with more efficient FDG metabolism.

At more severe levels of amyloid pathology, the current results show a positive relationship between FDG metabolism and the residual reserve index, which suggests that individuals with a higher residual reserve index are expected to have higher downstream executive function performance due to a compensatory upregulation of metabolic activity. We posit that these results are consistent with the concept of neural metabolic capacity, as they suggest that a higher residual reserve index benefits cognitive performance by allowing healthy neurons to increase their metabolic activity in the face of neurological insult. Taken together, the results of our study suggest that the variance in the residual reserve index is capturing variation in cognitive reserve (Barulli and Stern, 2013): at average and below levels of amyloid pathology, the residual reserve index manifests as variation in metabolic efficiency, and at high levels of amyloid pathology, the residual reserve index manifests as variation in metabolic capacity.

### Strengths, limitations, and future directions

One salient limitation of our study is that, while we have interpreted our results under the assumption that the residual reserve index is modifying the effect of amyloid pathology on FDG metabolism, we cannot exclude the reverse possibility because amyloid pathology was measured at the same time point as the residual reserve index; some argue a moderator should be modeled as antecedent to the effects being modified (Kraemer et al., 2008). It was not possible for us to model the residual reserve index and amyloid pathology at different time points within the overall sequential mediation model due to insufficient data coverage. Future research that can compare the results of a model using the residual reserve index as the initial antecedent, to one in which amyloid pathology is the initial antecedent may be able to characterize their interaction with greater clarity, e.g., such a study may be able to test Jagust and Mormino’s (2011) hypothesized negative association between cognitive reserve and one’s degree of amyloid accumulation.

A strength of this study is that we included baseline measurements of AT(N) biomarkers in the memory performance decomposition model that created the residual reserve index, in contrast to prior iterations of the residual reserve index that accounted only for structural brain integrity (Reed et al., 2010; McKenzie et al., 2020). The AT(N) biomarkers, particularly the amyloid and tau biomarkers, are more sensitive to early neurodegenerative change than MRI indicators of structural brain integrity, and likely added meaningful information about baseline memory performance (Hohman et al., 2016). By adding biomarkers of Alzheimer’s disease pathology and neurodegeneration as predictors in the decomposition model, we may also have accounted for some variance in memory performance that relates to brain maintenance (Cabeza et al., 2018). In other words, adding the AT(N) biomarkers to the decomposition model may have resulted in a more precise indicator of cognitive reserve. However, it also means that the residual reserve index created in our study is not directly comparable to the residual reserve index used in prior studies, as the interpretation of the residual reserve index depends on the predictors used in the decomposition (Ewers, 2020). This could mean that the residual reserve index used in the current study may have captured a unique facet of cognitive reserve compared to other versions of the residual reserve index. This idea could be examined in a replication of our study that compares the results using our residual reserve index vs. a different version of the residual reserve index, such as one defined using only indicators of structural brain integrity (per the original model by Reed et al., 2010).

Another strength is our use of a sequential mediation model, which allowed us to exclude the possibility of bidirectional relationships between biomarkers; this was especially important given some research suggests that metabolic activity influences the accumulation of amyloid pathology (Cohen et al., 2009; Jagust and Mormino, 2011). In addition, the current study revealed that the association between a CSF biomarker of amyloid pathology and downstream FDG metabolism, independent from plasma tau pathology, comprises a substantial proportion of the overall effect of amyloid pathology on future executive function performance; further, we demonstrated that the residual reserve index preferentially moderated this association rather than all other possible paths in our model of the amyloid cascade. Perhaps further understanding of cognitive resilience mechanisms can be gleaned by studying the mechanisms by which amyloid pathology affects FDG metabolism, and by identifying factors that modify the expression of these mechanisms.

In a similar vein, future research could build on our findings by examining changes in amyloid pathology and FDG metabolism over time, to quantify the degree of amyloid pathology needed to trigger compensatory increases in FDG metabolism predicted by the residual reserve index, and whether this threshold varies with the residual. In addition, our findings may be extended by replicating our analyses using individual FDG regions of interest, as this may reveal important information about possible cognitive resilience mechanisms; for example, FDG metabolism in prefrontal regions may be associated with cognitive reserve (e.g., Morbelli et al., 2013). Modeling dynamic change in the residual reserve index may also provide valuable information in future studies that aim to extend on our findings, as the rate of depletion of the residual may be an important predictor of future outcomes (Bettcher et al., 2019). A longitudinal mediation model would have the advantage of controlling for autoregressive effects in the predictors (Cain et al., 2018), which was not possible with our sequential mediation model.

Given that the residual reserve index contains variance in memory performance which is “unexplained,” another important step in extending upon our findings would be to examine associations between the residual reserve index and modifiable lifestyle factors purported to build cognitive reserve (e.g., engagement in cognitively stimulating activities over the lifespan). Factors strongly associated with the residual reserve index may then be tested within the AT(N) framework to identify potential mechanisms underlying cognitive resilience to Alzheimer’s disease pathology, and highlight important targets for interventions that aim to reduce the risk of dementia due to Alzheimer’s disease.

## Conclusion

The current study presents a novel investigation of the moderating effects of the residual reserve index on future executive function in the context of a sequential mediation model based on the modified amyloid cascade hypothesis (Jack et al., 2013). We found significant indirect pathways from CSF amyloid to executive function outcomes via tau pathology and FDG metabolism in series, and via FDG metabolism independent from tau, the latter moderated by the residual reserve index. Our results showed that the relationship between FDG metabolism and future executive function performance varies as a function of the interaction between antecedent amyloid pathology and the residual reserve index. At less severe levels of amyloid pathology, individuals with a higher residual reserve index are expected to show lower FDG metabolism, but a comparable future executive function performance, relative to those with a lower residual reserve index. When amyloid pathology is more severe, a higher residual reserve index benefits future executive function performance, via an association with higher FDG metabolism. We propose that these effects of the residual reserve index are consistent with the cognitive resilience conferred by cognitive reserve, insofar as cognitive reserve increases metabolic efficiency and the capacity of neurons to increase metabolism to compensate for neurodegeneration and maintain cognitive function in the face of greater amyloid burden.

## Data availability statement

The data analyzed in this study is subject to the following licenses/restrictions: Data is available from ADNI upon request. Requests to access these datasets should be directed to https://adni.loni.usc.edu/data-samples/access-data/.

## Ethics statement

Ethical review and approval was not required for the study on human participants in accordance with the local legislation and institutional requirements. The patients/participants provided their written informed consent to participate in this study.

## Author contributions

CM was responsible for study design, data analysis and interpretation of results, and was the primary contributor in writing the manuscript. RB assisted with data analysis and interpretation of results and contributed feedback and revisions to the written manuscript. PB contributed to data preparation, as well as feedback and revisions to the written manuscript. MW and OS contributed feedback and revisions to the written manuscript. BG assisted with study design, data analysis and interpretation of results, and contributed feedback and revisions to the written manuscript. All authors have read and approved the final manuscript.

## Footnotes

1
http://surfer.nmr.mgh.harvard.edu/

## Abbreviations

Aβ_42_/Aβ_40_: ratio of CSF β-amyloid_1–42_ to β-amyloid_1–40_
ADNI-Mem: ADNI’s composite measure of episodic memory
ADNI-EF: ADNI’s composite measure of executive function
AT(N): the biological classification system wherein AD is classified according to the presence/severity of amyloid pathology (A), tau pathology (T), and neurodegeneration (N)
HCV: bilateral hippocampal volume
p-tau181: plasma phosphorylated tau 181
WBV: whole brain volume
WMH: white matter hyperintensity volume
TIV: total intracranial volume.
