# Supplementary material for: Residual reserve index modifies the effect of amyloid pathology on fluorodeoxyglucose metabolism: Implications for efficiency and capacity in cognitive reserve
Source: Front Aging Neurosci. 2022 Aug 12;14:943823. doi: 10.3389/fnagi.2022.943823 (PMC9413056; doi:10.3389/fnagi.2022.943823)
Supplement: Supplementary file 1 [file Data_Sheet_1.pdf]

Supplementary Material

S1. Methods

S1.1. White Matter Hyperintensity Volumes

Pre-processed WMH volumes were downloaded directly from the ADNI database. T2-weighted FLAIR scans were performed on ADNI2 participants using 3T scanners, and WMH volumes were estimated using a Bayesian approach. Details about ADNI's FLAIR acquisition and WMH estimation procedures are reported by Scott et al. (2015). The distribution of WMH volumes was strongly positively skewed; therefore, it was log-transformed before analyses.

S1.2. FDG-PET Data

A measure of the mean FDG standard uptake value ratio (SUVR) across five meta-regions of interest (meta-ROIs) was used as a measure of neurodegeneration (Jack et al., 2018). Details of ADNI's FDG PET acquisition methods are available at http://adni.loni.usc.edu/ and have been described previously (e.g., Landau et al., 2010). The FDG meta-ROIs were selected as the most cited regions in a meta-analysis of MCI and AD literature: the left and right angular gyri, left and right middle/inferior temporal gyri, and bilateral posterior cingulate cortex. The SUVR in each meta-ROI was normalised using the pons as a reference (Landau et al., 2010, 2011).

S1.3. CSF $A\beta_{42}/A\beta_{40}$

The ratio of β-amyloid$_{1\text{-}42}$ to β-amyloid$_{1\text{-}40}$ ($A\beta_{42}/A\beta_{40}$) in CSF was used as the measure of amyloid pathology (Jack et al., 2018); lower $A\beta_{42}/A\beta_{40}$ values indicate greater amyloid pathology. Baseline (T0) $A\beta_{42}/A\beta_{40}$ measurements were downloaded directly from the ADNI database. The ADNI CSF data collection and $A\beta_{42}/A\beta_{40}$ analysis methods have been detailed previously (Korecka et al., 2014; Kang et al., 2015). Measurement of CSF $A\beta_{42}$ and $A\beta_{40}$ concentration was completed at the University of Pennsylvania by the ADNI Biomarker Core, using a two-dimensional ultra-performance liquid chromatography tandem mass spectrometry platform; $A\beta_{42}$ results were validated against the well-established Elecsys $A\beta_{42}$ immunoassay (AlzBio3).

S1.4. Plasma p-tau181

Tau pathology was measured using plasma phosphorylated tau 181 (p-tau181; Jack et al., 2018). Data from baseline (T0) and the first annual follow-up (T1) were downloaded directly from the ADNI database to be used in the decomposition and moderated sequential mediation models, respectively. Concentrations of plasma p-tau181 were measured at the Clinical Neurochemistry Laboratory, University of Gothenburg (Mölndal, Sweden), on a Simoa HD-X (Quanterix) instrument, as reported in detail by Karikari and colleagues (2020). We considered using CSF p-tau181 as our tau biomarker, but decided to use plasma to capitalize on the larger sample size at T1 (per ADNI protocol; see Kang et al., 2015).

S1.5. Statistical Analyses

S1.5.1. Validity of the MEMR Plausible Values

In addition to estimating the reliability of the MEMR plausible values obtained via Bayesian SEM, validity was evaluated using a longitudinal growth model of ADNI-EF. For the purpose of this analysis, the ADNI-EF intercept was estimated at T0. The MEMR latent variable was entered as a predictor of ADNI-EF intercept and slope, with other variables from the decomposition model entered as covariates, in a single simultaneous model (Figure S1A). Next MEMR and covariates were used to predict ADNI-EF intercept and slope in a separate model, using the saved MEMR plausible values in place of the latent variable (Figure S1B); these results were aggregated over 30 imputed datasets. Consistency between the parameter estimates was judged based on whether the absolute and relative magnitudes of the estimates looked similar between the two models.

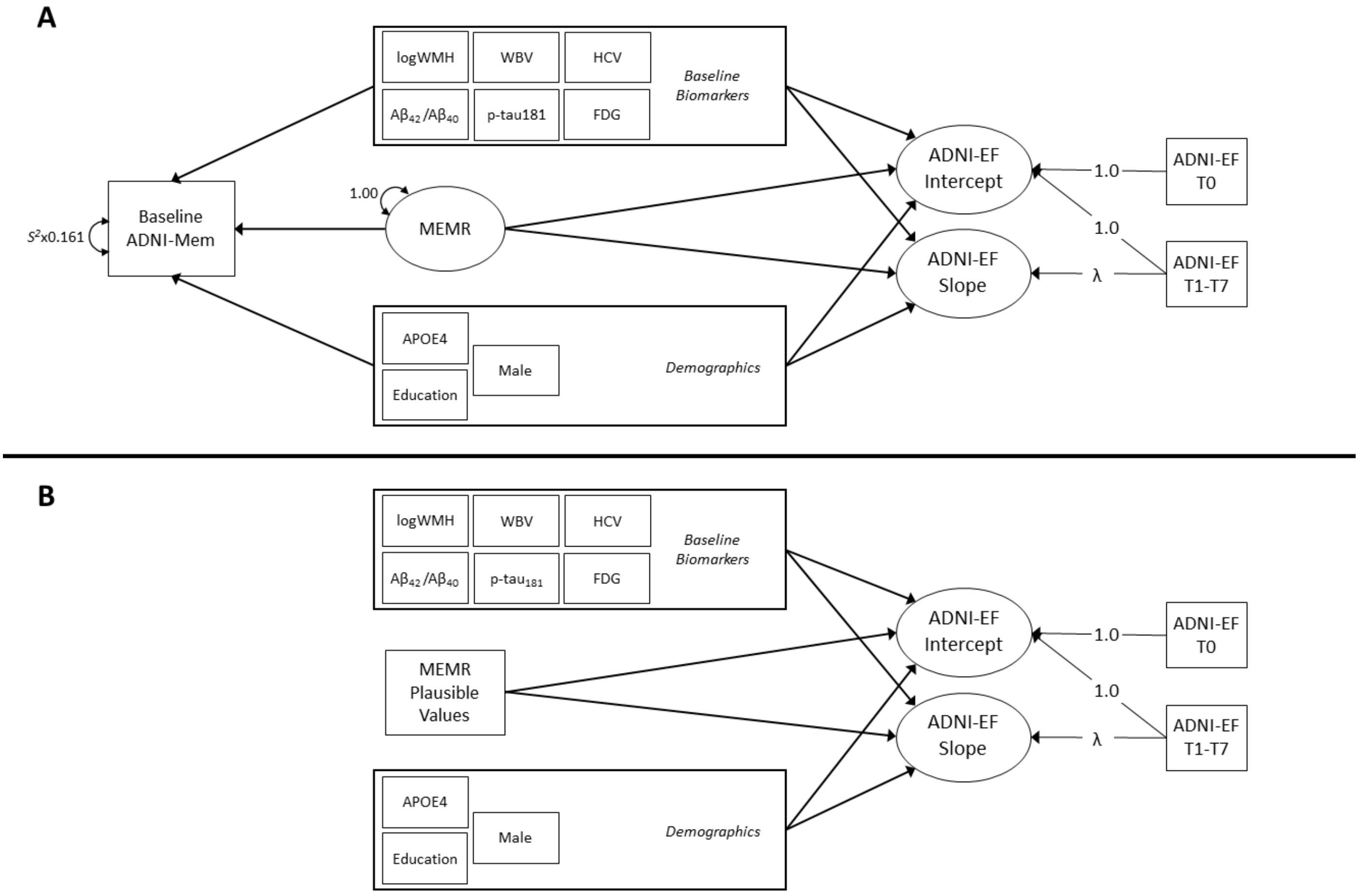

Figure S1. The longitudinal growth models used to evaluate the validity of the MEMR plausible values. Rectangles denote observed variables and ovals represent latent variables. Baseline biomarkers and demographics are enclosed in boxes to simplify interpretation. ADNI-EF intercept is defined by the baseline visit (T0). λ denotes the factor loadings used to define a linear slope over seven years (T1 to T7). Not shown: whole brain (WBV) and bilateral hippocampal (HCV) volumes were regressed onto total intracranial volume to correct for head size. **(A)** the model using the latent variable to predict ADNI-EF intercept and slope (i.e., LGM tested simultaneously with the ADNI-Mem decomposition). Correlations between observed variables were initially freely estimated, then non-significant correlations were constrained to zero to improve model fit (Reed et al., 2010). **(B)** the model using saved plausible values, obtained via Bayesian SEM, in place of the MEMR latent variable. MEMR plausible values were entered as observed data and parameter estimates were aggregated using results from 30 imputed datasets. Abbreviations: MEMR, the residual reserve index; ADNI-Mem, ADNI's composite of episodic memory performance; ADNI-EF, ADNI's composite of executive function performance; logWMH, log-transformed white matter hyperintensity volume; $A\beta_{42}/A\beta_{40}$, ratio of CSF β-amyloid$_{1-42}$ to β-amyloid$_{1-40}$;p-tau$_{181}$, plasma phosphorylated tau 181; FDG, uptake of ^{18F}fluorodeoxyglucose tracer in AD-specific regions of interest; APOE4, number of apolipoprotein-E ε4 alleles.

S1.5.2. Longitudinal growth of ADNI-EF

The latent growth factors (intercept and linear slope) of ADNI-EF are shown in Figure S1; these growth factors were the distal outcomes in the moderated mediation model used to test this study's hypotheses, and were estimated simultaneously within the moderated sequential mediation model (Figure 1B in main text). The ADNI-EF intercept was estimated at T3 so it would be the temporal consequence of the predictors and mediators in the model, which were measured consecutively at T0 (amyloid and the residual reserve index), T1 (tau), and T2 (FDG metabolism).

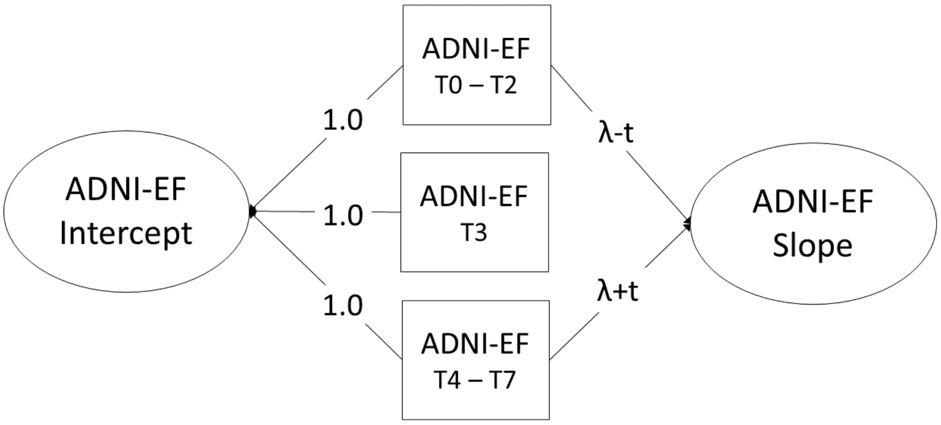

Figure S2. Diagram showing the estimation of ADNI-EF intercept and slope. The ADNI-EF intercept and slope were defined using data collected from baseline (T0) through to the seventh annual follow-up (T7). The intercept was defined by ADNI-EF scores at the third annual follow-up (T3). λ-t denotes negative factor loadings for ADNI-EF measurements taken prior to T3, and λ+t denotes positive factor loadings for ADNI-EF measurements taken after T3.

S2. Results

S2.1. Comparing ML and Bayesian Decomposition Models

Table S1 shows the parameter estimates obtained from analysing the decomposition model (Figure 2 in main text) using ML and Bayesian estimation. The parameter estimates in the Bayesian decomposition model were identical or near-identical to the ML-derived model.

Table S1. Standardised parameter estimates from the ML and Bayesian decomposition models.

		ML Estimation		Bayesian Estimation	
		**β**	**SE**	**β**	**Posterior SD**
Factor loading					
MEMR by	ADNI-Mem	0.564**	0.014	0.563**	0.015
Regression coefficients					
ADNI-Mem on	Male	-0.191**	0.020	-0.191**	0.020
	Education	0.159**	0.017	0.159**	0.017
	HCV	0.350**	0.027	0.351**	0.027
	WBV	-0.075*	0.028	-0.074*	0.028
	logWMH	-0.023	0.023	-0.024	0.023
	$A\beta_{42}/A\beta_{40}$	0.174**	0.031	0.173**	0.031
	p-tau_{181}	0.022	0.024	0.022	0.024
	FDG	0.331**	0.023	0.331**	0.023
	APOE4	-0.043[a]	0.023	-0.044*	0.023
Residual Variance					
	ADNI-Mem	0.162**	0.005	0.161**	0.006

[a]p=0.060

S2.2. Validity of the MEMR Plausible Values

Table S2 shows the parameter estimates from the two LGMs shown in Figure S1. Fit was good for the model using the MEMR latent variable, RMSEA = 0.032, 90% CI [0.028 – 0.036]; CFI = 0.985; TLI = 0.979; SRMR = 0.027, and the model using plausible values, RMSEA = 0.031, 90% CI [0.027 – 0.035]; CFI = 0.983; TLI = 0.979; SRMR = 0.041. The parameter estimates for the covariates were similar in magnitude across both models. The MEMR latent variable had a stronger regression effect on ADNI-EF intercept and slope compared to the MEMR plausible values, but the effect of MEMR was significant and positive in both models. Overall, these results indicated that the saved MEMR plausible values were valid to use in place of the MEMR latent variable in the moderated sequential mediation reported in the main text.

Table S2. Standardised parameter estimates obtained from LGMs using MEMR latent variable versus imputed plausible values.

		MEMR Latent Variable (Figure S1A)		MEMR Plausible Values[a] (Figure S1B)	
Outcome	Predictor	β	SE	β	SE
ADNI-EF Intercept	HCV	0.136**	0.030	0.147**	0.029
	WBV	0.186**	0.031	0.192**	0.032
	logWMH	-0.124**	0.024	-0.139**	0.026
	$A\beta_{42}/A\beta_{40}$	0.122**	0.030	0.109**	0.029
	p-tau$_{181}$	0.034	0.027	0.035	0.028
	FDG	0.426**	0.024	0.424**	0.024
	Male	-0.148**	0.022	-0.154**	0.024
	Educ	0.175**	0.018	0.179**	0.018
	MEMR	0.407**	0.037	0.310**	0.020
ADNI-EF slope	HCV	0.256**	0.051	0.248**	0.053
	WBV	-0.137*	0.054	-0.124*	0.057
	logWMH	-0.033	0.043	-0.048	0.045
	$A\beta_{42}/A\beta_{40}$	0.307**	0.043	0.295**	0.045
	p-tau$_{181}$	-0.124*	0.059	-0.130*	0.061
	FDG	0.372**	0.042	0.368**	0.043
	Male	0.083*	0.038	0.072	0.040
	Education	-0.029	0.031	-0.033	0.032
	MEMR	0.268**	0.037	0.215**	0.037

Note. Parameter estimates for MEMR plausible values are averages from the 30 datasets that were imputed using Bayesian estimation; SEs are computed using the average SEs from the 30 datasets and the between analysis parameter estimate variation (Schafer, 1997; Muthén and Muthén, 2017). Abbreviations: β, standardised coefficient; SE, standard error; HCV, bilateral hippocampal volume; WBV, whole brain volume; ADNI-EF, ADNI's composite of executive function performance; logWMH, log-transformed white matter hyperintensity volume; $A\beta_{42}/A\beta_{40}$, ratio of CSF β-amyloid$_{1\text{-}42}$ to β-amyloid$_{1\text{-}40}$; p-tau$_{181}$, plasma phosphorylated tau 181; FDG, uptake of ^{18F}fluorodeoxyglucose tracer in AD-specific regions of interest; MEMR, the residual reserve index.

S2.3. Post-Hoc Analysis: Cortical Amyloid Deposition vs CSF Amyloid Concentration

We considered the possibility that our findings may have been different had we used a direct measure of cortical amyloid burden at baseline, as the concordance between CSF $A\beta_{42}/A\beta_{40}$ and PET measures of amyloid deposition is high but imperfect (e.g., Lewczuk et al., 2017). Pre-processed T0 summaries of cortical florbetapir (AV45) uptake (e.g., Chen et al., 2015) were downloaded directly from the ADNI database and used in place of CSF $A\beta_{42}/A\beta_{40}$ in the moderated sequential model (Figure 1B in the main text). All other aspects of the moderated sequential model were identical to the model described in the main text. The results

of this secondary analysis, reported in Figure S3, were consistent with the primary findings reported in the main text. Fit was good: RMSEA = 0.032, 90% CI [0.018 0.044]; CFI = 0.979; TLI = 0.977; SRMR = 0.051.

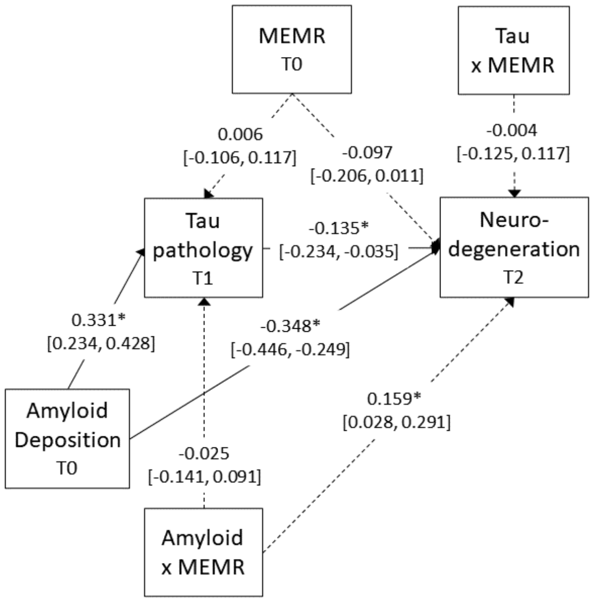

		ADNI-EF Intercept (T3)	ADNI-EF Slope (T0 - T7)
Total Effect	Amyloid Deposition	-0.458*<br>[-0.563, -0.353]	-0.080*<br>[-0.100, -0.060]
Direct Effects	Amyloid Deposition	-0.350*<br>[-0.320, -0.127]	-0.057*<br>[-0.078, -0.037]
	MEMR	0.288*<br>[0.188, 0.389]	0.007<br>[-0.013, 0.028]
	Amyloid x MEMR	-0.010<br>[-0.108, 0.128]	0.004<br>[-0.024, 0.033]
	Tau Pathology	-0.016<br>[-0.131, 0.071]	0.000<br>[-0.017, 0.017]
	Tau x MEMR	-0.020<br>[-0.118, 0.079]	0.000<br>[-0.019, 0.020]
	Neurodegeneration	0.584*<br>[0.486, 0.682]	0.058*<br>[0.036, 0.081]
	Neurodegeneration x MEMR	-0.015<br>[-0.141., 0.110]	0.002<br>[-0.024, 0.028]
Indirect Effects	B_{AT}	-0.005<br>[-0.032, 0.020]	0.000<br>[-0.006, 0.005]
	B_{ATN}	-0.027*<br>[-0.047, -0.005]	-0.003*<br>[-0.005, -0.001]
	B_{AN}	-0.203*<br>[-0.288, -0.116]	-0.028*<br>[-0.032, -0.008]

Figure S3. Parameter estimates from the moderated sequential mediation model using amyloid deposition, measured using AV45 PET, as the biomarker of amyloid pathology. $N = 362$. Parameter estimates that are significant at the $p < .05$ level are marked with *. The 95% CIs for the indirect effects (bottom-right of figure) are bias-corrected bootstrapped confidence intervals (2000 draws), obtained from a model estimated using the average residual reserve index (MEMR) plausible values. All other 95% CIs are symmetric intervals (±1.96 standard errors) obtained by pooling results from 30 imputed datasets. Dashed lines are used for illustrative purposes, to signify paths that relate to the moderating effect of MEMR; solid lines relate to mediation relationships. Biomarkers used were the cortical uptake of florbetapir (AV45) for amyloid deposition; plasma phosphorylated tau 181 (p-tau181) for tau pathology; and uptake of ^{18F}fluorodeoxyglucose tracer (FDG metabolism) in AD-specific regions of interest for neurodegeneration. ADNI-EF denotes ADNI's composite measure of executive function. Abbreviations: B_{AT}, the amyloid → tau → ADNI-EF indirect effect; B_{ATN}, the amyloid → tau → neurodegeneration → ADNI-EF indirect effect; B_{AN}, the amyloid → neurodegeneration → ADNI-EF indirect effect.

References

Chen, K., Roontiva, A., Thiyyagura, P., Lee, W., Liu, X., Ayutyanont, N., et al. (2015). Improved power for characterizing longitudinal amyloid-βPET changes and evaluating amyloid-modifying treatments with a cerebral white matter reference region. *J. Nucl. Med.* 56, 560–566. doi:10.2967/jnumed.114.149732.

Jack, C. R., Bennett, D. A., Blennow, K., Carrillo, M. C., Dunn, B., Haeberlein, S. B., et al. (2018). NIA-AA Research Framework: Toward a biological definition of Alzheimer's disease. *Alzheimer's Dement.* 14, 535–562. doi:10.1016/j.jalz.2018.02.018.

Kang, J. H., Korecka, M., Figurski, M. J., Toledo, J. B., Blennow, K., Zetterberg, H., et al. (2015). The Alzheimer's Disease Neuroimaging Initiative 2 Biomarker Core: A review of progress and plans. *Alzheimer's Dement.* 11, 772–791. doi:10.1016/j.jalz.2015.05.003.

Karikari, T. K., Pascoal, T. A., Ashton, N. J., Janelidze, S., Benedet, A. L., Rodriguez, J. L., et al. (2020). Blood phosphorylated tau 181 as a biomarker for Alzheimer's disease: a diagnostic performance and prediction modelling study using data from four prospective cohorts. *Lancet Neurol.* 19, 422–433. doi:10.1016/S1474-4422(20)30071-5.

Korecka, M., Waligorska, T., Figurski, M., Toledo, J. B., Arnold, S. E., Grossman, M., et al. (2014). Qualification of a surrogate matrix-based absolute quantification method for amyloid-β_{42} in human cerebrospinal fluid using 2D UPLC-tandem mass spectrometry. *J. Alzheimers. Dis.* 41, 441–451. doi:10.3233/JAD-132489.

Landau, S. M., Harvey, D., Madison, C. M., Koeppe, R. A., Reiman, E. M., Foster, N. L., et al. (2011). Associations between cognitive, functional, and FDG-PET measures of decline in AD and MCI. *Neurobiol. Aging* 32, 1207–1218. doi:10.1016/j.neurobiolaging.2009.07.002.

Landau, S. M., Harvey, D., Madison, C. M., Reiman, E. M., Foster, N. L., Aisen, P. S., et al. (2010). Comparing predictors of conversion and decline in mild cognitive impairment. *Neurology* 75, 230–238. doi:10.1212/WNL.0b013e3181e8e8b8.

Lewczuk, P., Matzen, A., Blennow, K., Parnetti, L., Molinuevo, J. L., Eusebi, P., et al. (2017).

Cerebrospinal Fluid Aβ42/40 Corresponds Better than Aβ42 to Amyloid PET in Alzheimer's Disease. *J. Alzheimer's Dis.* 55, 813–822. doi:10.3233/JAD-160722.

Muthén, L. K., and Muthén, B. O. (2017). *MPlus User's Guide*. Eighth. Los Angeles, CA: Muthén & Muthén Available at: www.StatModel.com [Accessed October 26, 2018].

Reed, B. R., Mungas, D., Farias, S. T., Harvey, D., Beckett, L., Widaman, K., et al. (2010). Measuring cognitive reserve based on the decomposition of episodic memory variance. *Brain* 133, 2196–2209. doi:10.1093/brain/awq154.

Schafer, J. L. (1997). *Analysis of incomplete multivariate data*. CRC Press.

Scott, J. A., Braskie, M. N., Tosun, D., Thompson, P. M., Weiner, M., DeCarli, C., et al. (2015). Cerebral Amyloid and Hypertension are Independently Associated with White Matter Lesions in Elderly. *Front. Aging Neurosci.* 7, 221. doi:10.3389/fnagi.2015.00221.
